# Sex differences in the associations of accelerometer-determined physical activity with physical and cognitive function in older adults living in long-term care

**DOI:** 10.3389/fpubh.2024.1446286

**Published:** 2024-11-11

**Authors:** Ziwei Zeng, Chun Liang Hsu, Kimberley Stefanie van Schooten, Yijian Yang

**Affiliations:** ^1^Department of Sports Science and Physical Education, The Chinese University of Hong Kong (CUHK), Shatin, Hong Kong SAR, China; ^2^Department of Rehabilitation Sciences, The Hong Kong Polytechnic University, Kowloon, Hong Kong SAR, China; ^3^Neuroscience Research Australia, University of New South Wales, Sydney, NSW, Australia; ^4^CUHK Jockey Club Institute of Aging, The Chinese University of Hong Kong, Shatin, Hong Kong SAR, China

**Keywords:** older adult, long-term care, sex, physical activity, cognition

## Abstract

Older adults residing in long-term care often experience declines in physical and cognitive functions despite the access to in-house physical activity (PA) programs. This study aimed to evaluate the associations of PA with physical function and global cognitive function in older adults living in long-term care, while examining potential sex differences. A cross-sectional analysis of baseline data from a two-arm cluster randomized controlled trial was conducted, involving 67 participants (34 men, 33 women). PA levels were assessed using tri-axial accelerometers. Physical function, including muscular strength, postural sway, and Short Physical Performance Battery (SPPB) and cognitive function were measured. Spearman correlation analysis revealed no significant associations between PA metrics and muscular strength, postural sway, or global cognitive function across the entire samples (*p* ≥ 0.091). Multiple linear regression models were developed for the entire sample, males, and females to examine the associations between PA and physical function measures and global cognitive function. After adjustments for confounders, light PA was significantly associated with higher SPPB sub-scores (gait: *β* = 0.600, *p* < 0.001; sit-to-stand: *β* = 0.574, *p* < 0.001), faster usual gait speed (*β* = 0.659, *p* = 0.012), and shorter sit-to-stand times (*β* = −0.305, *p* = 0.041) across the whole sample. Similar significant associations were observed in males between light PA and SPPB scores (total: *β* = 0.319, *p* = 0.040; gait: *β* = 0.532, *p* < 0.001; sit-to-stand: *β* = 0.417, *p* = 0.009), usual gait speed (*β* = 0.762, *p* = 0.017), and sit-to-stand times (*β* = −0.677, *p* < 0.001). In females, a significant association was found between light PA and global cognitive function (*β* = 0.319, *p* = 0.012) after adjusting for confounders. This study highlights sex differences in the association of accelerometer-determined PA with physical and cognitive function in older adults living in long-term care, with LPA showing beneficial effects, especially for physical function in males and for cognitive function in females.

## 1 Introduction

The global population aged 60 and over is rapidly increasing (1), with projections indicating that by 2050, it will comprise 21.1% of the entire global population (2). This shift in the proportion of older adults is accompanied by increased prevalence of aging-related pathologies and disabilities, thereby affecting their daily functioning (3). Those with limited self-care abilities due to poorer health often choose long-term care to access 24-h professional care (4). However, residents in these facilities frequently experience progressive declines in both physical and cognitive functions (5).

Physical impairments often manifest early in individuals with cognitive decline, and mobility decline is predictive of disabilities in older adults (6). Therefore, it is important to assess physical function in older adults with emphasis on mobility, balance, and muscular strength, given that these are key correlates of cognitive changes (7, 8). As physical function deteriorates, older adults may limit their participation in instrumental activities of daily living (ADLs), leading to a sedentary lifestyle and an increased risk for dependency, healthcare needs, and diminished quality of life (QoL) (9–11). Cognitive impairment further exacerbates this situation by increasing the risk of dementia progression and adding burdens to families and society (12–14). It is a major risk factor for institutionalization and the need for long-term care.

Previous research highlights the positive effects of physical activity (PA) on both physical and cognitive health in older adults living in long-term care (15–18). However, the specific relationship between objectively measured PA levels and physical function and cognitive performance in this population remains underexplored. Recent studies have utilized tri-axial accelerometers to accurately evaluate PA, which is commonly categorized into sedentary behavior (SB; low-intensity activities with a Metabolic Equivalent of Task [MET] of <1.5), light PA (LPA, activities with a MET of 1.5–2.9), and moderate-to-vigorous intensity PAs (MVPA, activities with a MET of ≥ 3.0) (19, 20). While the World Health Organization recommends at least 150 min of MVPA weekly for older adults (21), achieving this can be challenging for frail individuals in long-term care (22). SB is associated with various negative health outcomes, including cardiovascular disease (23), falls (24), frailty (25), and mortality (26).

Despite PA programs are readily available in care facilities, older adults residing in long-term care exercise less (0.2 h per day, or 84 min per week of MVPA) and are more sedentary (9.2 h per day of SB) compared to their community-dwelling counterparts (27–29). The severity of physical and cognitive deficits in this population, combined with limited caregiver monitoring, reduces the accuracy of self-reported or caregiver-reported PA levels (30). Therefore, objective measurements of PA and their relationship with physical and cognitive functions in frail older adults could provide insights into the declines in PA and help develop interventions to improve QoL and survival time.

Furthermore, compared to men, women are generally less physically active and have weaker muscular strength for those dwelling in the community and long-term care (31–33). Sex differences in PA levels and physical function are well-documented in older adults, but it is unclear if these differences extend to the correlations between PA levels and physical function performance and global cognitive function in long-term care residents. Understanding these sex differences is crucial for developing tailored interventions. Men and women may respond differently to PA due to various biological, psychological, and social factors. Tailoring PA programs to address these differences could enhance their effectiveness, potentially improving physical and cognitive outcomes and QoL for both sexes. Identifying these differences could also aim in designing more effective health policies and resource allocation, ensuring that both men and women receive optimal care and support.

The study aimed to evaluate the associations between PA levels and both physical function and global cognitive function in older adults residing in long-term care. A secondary objective was to examine sex differences in these associations. We hypothesized that more time spent in SB would be associated with lower physical and cognitive performance in both men and women. By elucidating these relationships, our study may provide a better understanding of how specific PA level, such as SB and LPA, impact overall health in this vulnerable population, offering a foundation for designing tailored interventions that maximize physical and cognitive benefits for each sex group.

## 2 Materials and methods

### 2.1 Study participants

The current study is a cross-sectional, secondary analysis of baseline data from a two-arm cluster randomized controlled trial (RCT) (34). Baseline data were collected from 20 long-term care facilities in Hong Kong between September 2023 and December 2023. Inclusion criteria were as follows: (1) aged 65 years or older; (2) able to rise from a chair, with or without using armrests and stand for at least 20 s; (3) confirmed by a physician to be able to participate in the study; and (4) able to wear an accelerometer continuously for 7 days to measure PA. Exclusion criteria included: (1) inability to comprehend instructions; (2) inability to complete the exercise program due to medical conditions; and (3) legal blindness (34). The study adhered to the principles of the World Medical Association Declaration of Helsinki and Good Clinical Practice. All participants provided written informed consent, either personally or through a family member, prior to study enrolment. Ethics approval was obtained from the Research Ethics Board at the Chinese University of Hong Kong and the Joint CUHK-NTEC Clinical Research Ethics Committee (34).

### 2.2 Outcome measures

#### 2.2.1 Physical activity level measurement

Participants’ PA levels were measured using a tri-axial accelerometer (ActiGraph GT9X Link IMU, Pensacola, FL, USA). The primary outcomes included the percentage of time spent in SB, LPA, and MVPA, and total step counts. Participants were instructed to wear the accelerometer on the right side of their waist for seven consecutive days, removing it only during bathing or before going to bed (35). Data were recorded in 1-min epochs at a sampling frequency of 100 Hz. Non-wearing time was defined as more than 60 consecutive minutes of inactivity (with activity intensity less than 1.0 METs), allowing up to 2 min of activity above 1.0 METs (35–38). A valid day was considered as at least 480 min (8 h) of wear time, because many participants required the assistance of a caregiver for proper wearing and charging of the accelerometer, taking into account the caregiver’s work hours. Data from at least 3 valid days analyzed using ActiLife software (39, 40). PA intensity was classified based on established cut-off points from studies on similar populations (41–43): SB, LPA, and MVPA levels were defined as 0–50 counts/min, 51–759 counts/min, and ≥760 counts/min, respectively. For this study, MVPA was categorized into bouts of activity lasting at least 3 min, since the participants were relatively frailer than their healthy counterparts (44).

#### 2.2.2 Physical function measurement

Physical function were objectively measured by trained research assistants. Measurements included maximum handgrip strength (right and left), upper limb strength (elbow flexion and extension), lower limb strength (knee extension), postural sway in the anterior–posterior (AP) and medial-lateral (ML) directions, and the Short Physical Performance Battery (SPPB) (34). Handgrip strength was measured using a digital hand-held dynamometry (HHD) (5001 Grip-A; Takei, Niigata City, Japan), with two measurements taken for each hand, and the maximum value recorded. Upper limb strength (biceps and triceps) and lower limb strength (quadriceps) were measured on the participant’s dominant side using the Hoggan microFET2 HHD (Hoggan Scientific, LLC, Salt Lake City, UT, USA). Two trails were performed for upper limbs, and three trials for lower limbs, with the maximum value used in analysis (45–47). Postural sway was measured in accordance with the Physiological Profile Assessment, with participant standing still on a hard surface with eyes open for 30 s (48). The AP and ML sway distances were recorded twice by the sway meter, and the average values were used for analysis. The SPPB included assessments of 4-m walking speed at self-selected speed (two measurements taken, with the fastest time recorded using a stopwatch), five-repetition sit-to-stand (STS) time, and 10 s standing balance in different positions (side-by-side, semi-tandem, and tandem). Each task was scored from 0 (lowest score) to 4 (highest score), with a total SPPB score ranging from 0 to 12 (49, 50). In this study, participants were allowed to use mobility aids for walking and to hold armrests when standing up from a chair if needed, considering their physical limitations.

#### 2.2.3 Cognitive function measurement

Global cognitive function was assessed using the Hong Kong version of the Montreal Cognitive Assessment (HK-MoCA), a validated tool widely utilized for assessing cognitive function in the Hong Kong population due to its accessibility and ease of administration (51, 52). The HK-MoCA evaluates seven cognitive domains, including visuospatial and executive function, naming, attention, language, abstraction, recall/short-term memory, and orientation. Both total and domain-specific scores were recorded, with a maximum score of 30 (Supplementary material 1).

#### 2.2.4 Other measurements

Several variables were considered potential confounders in the relationship between PA, physical function, and cognitive function, including age, sex, body mass index (BMI), education level, mobility aid used, frailty, and facility site. Body height and weight were measured by facility staff, and BMI was calculated. The mobility aids use was categorized into four levels: independent, cane-using, walker-using, and dependent. Frailty status was assessed using the 7-item FRAIL-NH scale, which evaluates fatigue, resistance, mobility, incontinence or disease, weight loss, eating style, and assistance with dressing. Each item was scored from 0 to 2, with a total score ranging from 0 (best) to 14 (worst) (34).

### 2.3 Statistical analyses

Statistical analyses were conducted using IBM SPSS (version 25.0, IBM SPSS Inc., Chicago, IL, USA), with significance level set at *p* < 0.05. Continuous variables were presented as mean ± standard deviation (SD) for normally distributed data, or median with interquartile range for non-normally distributed data. Categorical variables were represented as frequency and percentage. Normality of continuous variables was identified by the Shapiro–Wilk test. Among the physical function measures analyzed in this study, 11 continuous variables had at least one missing value, with an overall missing rate of 4.82%. Missing data were handled using multiple imputation. Descriptive statistics were calculated, and sex differences were tested using independent sample t-tests for normally distributed continuous variables, Mann–Whitney U tests for non-normally distributed continuous variables, and the chi-square test for categorical variables.

Bivariate Spearman correlation analyses (*r*) were conducted to explore the simple associations between objectively measured PA levels and physical function measurements and global cognitive scores for the total sample, using Matlab (version R2023b, MathWorks, Inc., Natick, MA, USA). Following *r* values were used to interpret the strength of correlation: 0–0.19 trivial; 0.20–0.39 weak; 0.40–0.59 moderate; 0.60–0.79 strong; and 0.80–1.00 very strong (53). Then, multiple linear regression models using the forward selection method were performed to examine the associations between PA metrics (independent variables) and physical test measures and global cognitive scores (dependent variables) for the total sample and for each sex group. Variables were retained in the final model if their *p*-values indicated statistical significance (*p* < 0.05). In the total sample models, sex was included as a covariate along with interaction terms to assess potential sex differences. Regardless of the significance of the interaction terms, we proceeded with separate models for males and females to explore potential sex-specific associations for all physical and cognitive outcomes. In sex-specific models, interaction terms were excluded, focusing on the main effects of PA metrics on the outcomes. Possible confounders, including age, BMI, education level, mobility aid used, frailty, and facility site, were included in all models. Multicollinearity of the independent variables was assessed by variance inflation factors (VIF), with values below five indicating no multicollinearity concerns. Beta coefficients were calculated to quantify the strength and direction of associations, representing the expected change in the dependent variable for each unit increase in the independent variables, holding other variables constant.

## 3 Results

### 3.1 Participant characteristics

**Figure 1:**
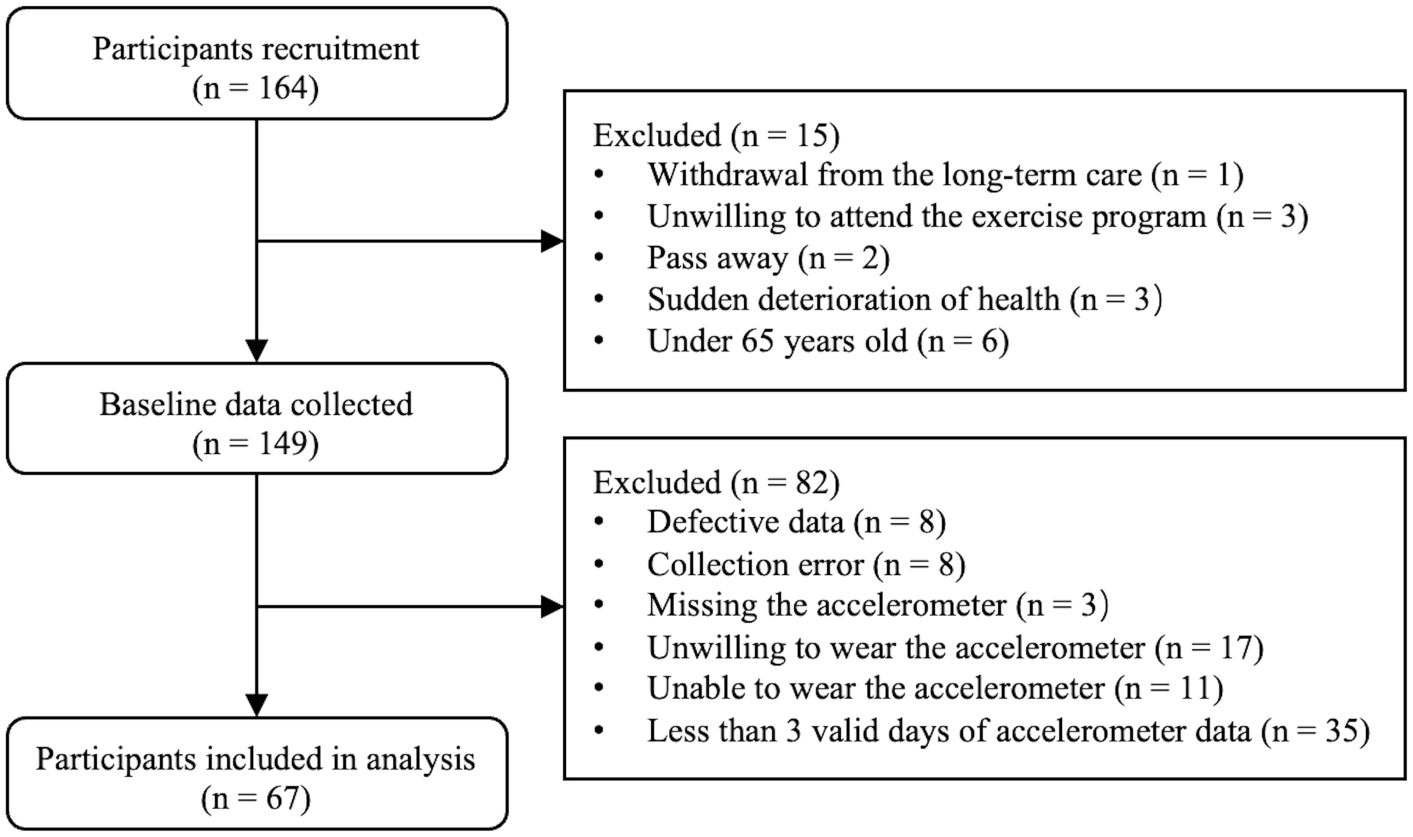
Flow chart of study participants.

Based on the inclusion criteria, the final analyzed population comprised 67 participants (34 men and 33 women), out of an initial recruitment pool of 164 participants (Figure 1). Descriptive characteristics for the entire sample and for each sex group are depicted in Table 1. Specifically, male participants were significantly taller, heavier, and exhibited greater upper and lower limb muscle strength. They also had better SPPB (total and sub-scores), faster STS performance, and higher domain-specific cognitive scores in attention and delayed recall compared to their female counterparts (*p* ≤ 0.046). However, there were no significant differences between men and women in age, BMI, education level, mobility aid used, PA metrics, body sway, usual gait speed, or total MoCA score (*p* ≥ 0.058).

**Table 1:** Characteristics of participants.

Variables	Total (*n* = 67)	Male (*n* = 34)	Female (*n* = 33)	*p* value
Age (years)	88.00 (14.00)	83.21 ± 9.29	90.00 (7.00)	0.064
Height (cm)	157.61 ± 9.53	163.91 ± 6.94	151.12 ± 7.21	**<0.001**
Weight (kg)	54.45 ± 10.64	58.79 ± 9.63	49.98 ± 9.85	**<0.001**
BMI (kg/m^2^)	21.94 ± 4.03	21.93 ± 3.60	21.96 ± 4.48	0.974
Education level				0.259
<11 years (n, %)	59 (88.06%)	28 (82.35%)	31 (93.94%)	
≥11 years	8 (11.94%)	6 (17.65%)	2 (6.06%)	
Mobility aid				0.231
Not used	23 (34.33%)	14 (41.18%)	9 (27.27%)	
Used	44 (65.67%)	20 (58.82%)	24 (72.73%)	
PA metrics				
SB (%)	86.12 (12.48)	85.68 (11.83)	84.61 ± 7.57	0.652
LPA (%)	10.14 (8.03)	11.00 ± 4.91	10.76 (9.45)	0.441
MVPA (%)	1.80 (2.40)	1.75 (2.87)	1.85 (2.60)	0.960
Step counts	1,995 (4,496)	1,995 (4,669)	1,914 (4,536)	0.573
Elbow flexion (kg)	7.29 ± 3.10	9.05 ± 3.04	5.48 ± 1.90	**<0.001**
Elbow extension (kg)	5.78 ± 2.09	6.89 ± 1.83	4.64 ± 1.72	**<0.001**
Knee extension (kg)	11.45 ± 4.16	12.80 ± 4.27	10.05 ± 3.59	**0.006**
Handgrip-R (kg)	13.50 (8.20)	17.24 ± 5.72	10.51 ± 3.29	**<0.001**
Handgrip-L (kg)	12.90 (8.80)	17.18 ± 4.95	10.05 ± 3.25	**<0.001**
Body sway-AP (mm)	22.57 (12.50)	22.75 (10.88)	22.57 (12.50)	0.363
Body sway-ML (mm)	35.00 (29.50)	31.50 (23.88)	36.50 (33.00)	0.350
SPPB total	5.00 (2.00)	5.82 ± 1.96	4.00 (2.00)	**<0.001**
Balance	2.00 (2.00)	3.00 (2.00)	2.00 (2.00)	**0.019**
Gait	1.00 (0.00)	1.00 (0.00)	1.00 (0.00)	**0.046**
STS	1.00 (0.00)	1.00 (1.00)	1.00 (0.00)	**<0.001**
Gait speed (m/s)	0.40 ± 0.18	0.43 ± 0.20	0.38 ± 0.16	0.246
5*STS time (s)	22.42 (14.15)	19.06 (13.13)	24.39 (12.45)	**0.013**
MoCA total	10.00 (13.00)	14.00 (15.00)	8.00 (10.00)	0.058
Visuospatial/executive	0.00 (2.00)	1.00 (2.00)	0.00 (1.00)	0.087
Naming	2.00 (2.00)	2.00 (2.00)	2.00 (3.00)	0.140
Attention	3.00 (4.00)	3.50 (3.00)	2.00 (3.00)	**0.020**
Language	2.00 (0.00)	2.00 (1.00)	2.00 (0.00)	0.734
Abstraction	1.00 (2.00)	1.00 (2.00)	0.00 (1.00)	0.127
Delayed recall	0.00 (1.00)	0.00 (3.00)	0.00 (0.00)	**0.021**
Orientation	3.00 (4.00)	3.00 (4.00)	2.00 (4.00)	0.144
FRAIL-NH	2.00 (3.00)	1.50 (2.00)	2.00 (4.00)	0.314

Continuous variables were presented as mean ± standard deviation (normal distribution) or median (interquartile range) (non-normal distribution); categorical variables were presented as frequency (percentage). Bold indicates statistical significance (*p* < 0.05); AP, anterior–posterior; BMI, body mass index; L, left; LPA, light physical activity; ML, medial-lateral; MoCA, Montreal Cognitive Assessment; MVPA, moderate-to-vigorous physical activity; PA, physical activity; R, right; SB, sedentary behavior; SPPB, Short Physical Performance Battery; STS, five-repetition sit-to-stand.

### 3.2 Correlations between physical activity metrics and physical function measurements and global cognitive function

**Figure 2:**
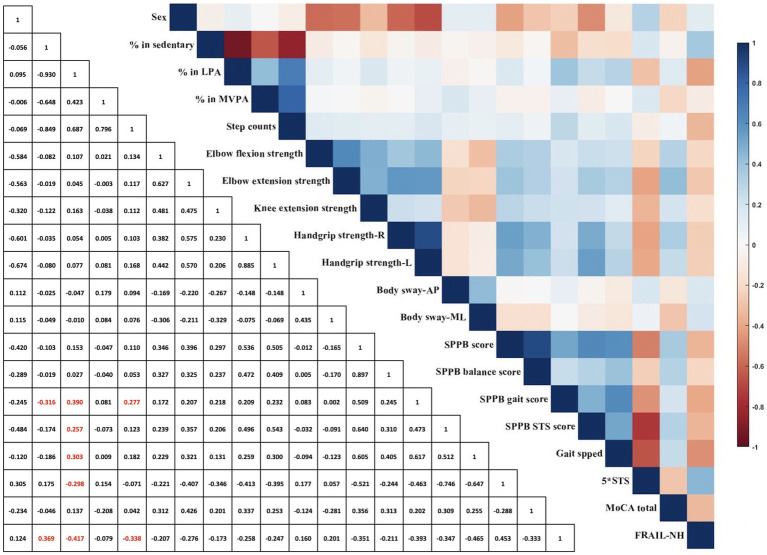
Spearman correlation coefficient between physical activity metrics, physical function measurements, and global cognitive function. AP, anterior–posterior; L, left; LPA, light physical activity; ML, medial-lateral; MoCA, Montreal Cognitive Assessment; MVPA, moderate-to-vigorous physical activity; R, right; SPPB, Short Physical Performance Battery; STS, five-repetition sit-to-stand; red color represents correlation is significant at the 0.05 level between physical activity metrics and physical function measurements (2-tailed).

Figure 2 illustrates the correlations between PA metrics and both physical and cognitive performance among older adults residing in long-term care. Significant but weak correlations were found between SB and both the SPPB gait score (*r* = −0.316, *p* = 0.009) and FRAIL-NH score (*r* = 0.369, *p* = 0.002). Weak correlations were also identified between LPA and the SPPB gait score (*r* = 0.390, *p* = 0.001) and STS score (*r* = 0.257, *p* = 0.035), usual gait speed (*r* = 0.303, *p* = 0.013), and STS time (*r* = −0.298, *p* = 0.014), while the FRAIL-NH score (*r* = −0.417, *p* = 0.009) had a significant moderate correlation with LPA. Step counts showed a significant weak correlation with both the SPPB gait score (*r* = 0.277, *p* = 0.023) and FRAIL-NH score (*r* = −0.338, *p* = 0.005). No significant correlations were identified between other PA metrics and physical function measures and total MoCA score (*p* ≥ 0.091).

The multiple linear regression analysis demonstrated that LPA was independently associated with physical function measurements. Specifically, in adjusted analyses, the percentage of time spent in LPA was significantly associated with the SPPB gait (*β* = 0.600, *p* < 0.001, adjusted *R*^2^ = 0.331) and STS score (*β* = 0.574, *p* < 0.001, adjusted *R*^2^ = 0.428), usual gait speed (*β* = 0.659, *p* = 0.012, adjusted *R*^2^ = 0.232), and STS time (*β* = −0.305, *p* = 0.041, adjusted *R*^2^ = 0.296). Notably, a significant interaction effect of sex was observed on the SPPB gait score (*β* = −0.425, *p* = 0.002) and STS score (*β* = −0.604, *p* < 0.001), and STS time (*β* = 0.319, *p* = 0.020). Other PA metrics were not significantly correlated with physical function measurements or global cognitive function in the total population. Full results are displayed in Table 2.

**Table 2:** Regression of percentage of light physical activity on physical function outcomes in total population (*n* = 67).

Dependent variables	Unadjusted					Adjusted				
	*B* (SE)	95% CI	*β*	*p* value	Adjusted *R*^2^	*B* (SE)	95% CI	*β*	*p* value	Adjusted *R*^2^
SPPB gait score	0.024 (0.007)	0.010–0.038	0.397	**<0.001**	0.145	0.037 (0.008)	0.021–0.053	0.600	**<0.001**	0.331
Sex * % in LPA						−0.020 (0.006)	−0.032 to −0.008	−0.425	**0.002**	
SPPB STS score	0.038 (0.016)	0.005–0.070	0.278	**0.023**	0.063	0.078 (0.016)	0.046–0.111	0.574	**<0.001**	0.428
Age						−0.022 (0.009)	−0.040 to −0.004	−0.240	**0.019**	
Sex * % in LPA						−0.063 (0.012)	−0.087 to −0.038	−0.604	**<0.001**	
Gait speed	0.010 (0.004)	0.003–0.018	0.325	**0.007**	0.092	0.021 (0.008)	0.005–0.037	0.659	**0.012**	0.232
Mobility aid used						−0.080 (0.026)	−0.133 to −0.027	−0.348	**0.004**	
5*STS	−0.453 (0.186)	−0.825 – −0.081	−0.289	**0.018**	0.069	−0.479 (0.229)	−0.936 to −0.021	−0.305	**0.041**	0.296
FRAIL-NH						1.114 (0.413)	0.288–1.941	0.312	**0.009**	
Sex * % in LPA						0.381 (0.160)	0.061–0.701	0.319	**0.020**	

Bold indicates statistical significance (*p* < 0.05); LPA, light physical activity; SE, standard error; SPPB, Short Physical Performance Battery; STS, sit-to-stand; 95% CI, 95% confidence interval. Unadjusted: only physical activity metrics as independent variables; adjusted: for confounders age, BMI, education level, mobility aid used, frailty, and facility site, and sex with its interaction terms with physical activity terms.

For male participants, similar associations were observed between LPA and physical function measurements. In the adjusted analysis, the percentage of LPA was independently associated with the SPPB score (total: *β* = 0.319, *p* = 0.040, adjusted *R*^2^ = 0.369; gait: *β* = 0.532, *p* < 0.001, adjusted *R*^2^ = 0.431; STS: *β* = 0.417, *p* = 0.009, adjusted *R*^2^ = 0.322), usual gait speed (*β* = 0.762, *p* = 0.017, adjusted *R*^2^ = 0.337), and STS time (*β* = −0.677, *p* < 0.001, adjusted *R*^2^ = 0.335). Full results are displayed in Table 3.

**Table 3:** Regression of percentage of light physical activity on physical function outcomes in male (*n* = 34).

Dependent variables	Unadjusted					Adjusted				
	*B* (SE)	95% CI	*β*	*p* value	Adjusted *R*^2^	*B* (SE)	95% CI	*β*	*p* value	Adjusted *R*^2^
SPPB total score	0.189 (0.060)	0.066–0.311	0.485	**0.004**	0.212	0.124 (0.058)	0.006–0.242	0.319	**0.040**	0.369
Age						−0.094 (0.031)	−0.158 to −0.030	−0.446	**0.005**	
SPPB gait score	0.053 (0.012)	0.029–0.077	0.619	**<0.001**	0.364	0.045 (0.012)	0.021–0.069	0.532	**<0.001**	0.431
Mobility aid used						−0.180 (0.082)	−0.348 to −0.012	−0.301	**0.036**	
SPPB STS score	0.101 (0.030)	0.040–0.162	0.513	**0.002**	0.240	0.082 (0.030)	0.022–0.142	0.417	**0.009**	0.322
Mobility aid used						−0.457 (0.207)	−0.879 to −0.034	−0.330	**0.035**	
Gait speed	0.015 (0.007)	0.002–0.029	0.376	**0.028**	0.115	0.031 (0.012)	0.006–0.056	0.762	**0.017**	0.337
Mobility aid used						−0.102 (0.044)	−0.190 to −0.013	−0.359	**0.027**	
5*STS	−0.949 (0.282)	−1.524 – −0.375	−0.511	**0.002**	0.238	−1.257 (0.294)	−1.856 to −0.658	−0.677	**<0.001**	0.335
Step counts						0.001 (0.000)	0.000–0.002	0.376	**0.024**	

Bold indicates statistical significance (*p* < 0.05); SE, standard error; SPPB, Short Physical Performance Battery; STS, sit-to-stand; 95% CI, 95% confidence interval. Unadjusted: only physical activity metrics as independent variables; adjusted: for confounders age, BMI, education level, mobility aid used, frailty, and facility site.

For female participants, in the adjusted analysis, the percentage of time spent in LPA was only independently associated with the total MoCA score (*β* = 0.319, *p* = 0.012, adjusted *R*^2^ = 0.552). Full results are displayed in Table 4.

**Table 4:** Regression of percentage of light physical activity on physical function and cognitive function outcomes in female (*n* = 33).

Dependent variables	Unadjusted					Adjusted				
	*B* (SE)	95% CI	*β*	*p* value	Adjusted *R*^2^	*B* (SE)	95% CI	*β*	*p* value	Adjusted *R*^2^
SPPB STS score	0.012 (0.004)	0.004–0.021	0.473	**0.005**	0.198	0.007 (0.003)	0.000–0.014	0.265	0.052	0.516
Education						0.435 (0.094)	0.243–0.627	0.605	**<0.001**	
MoCA total score	0.380 (0.164)	0.045–0.715	0.383	**0.028**	0.119	0.317 (0.118)	0.076–0.557	0.319	**0.012**	0.552
Age						−0.561 (0.101)	−0.767 to −0.355	−0.661	**<0.001**	

Bold indicates statistical significance (*p* < 0.05); MoCA, Montreal Cognitive Assessment; SE, standard error; SPPB, Short Physical Performance Battery; STS, sit-to-stand; 95% CI, 95% confidence interval. Unadjusted: only physical activity metrics as independent variables; adjusted: for confounders age, BMI, education level, mobility aid used, frailty, and facility site.

## 4 Discussion

This study provides valuable insights into the associations between objectively measured PA and physical and cognitive function in older adults living in long-term care. Previous studies have highlighted the positive impact of PA on these functions in older adults, aligning with our findings (54–57). However, the nuanced relationships, particularly in the context of long-term care and sex-specific differences, have not been thoroughly explored. Our study addresses this gap, revealing significant associations between LPA and various physical function measurements, as well as global cognitive function in this population. Notably, sex differences emerged in the strength of these associations. In male participants, LPA positively correlated with several mobility functions, including SPPB performance, usual gait speed, and time spent in STS tasks, even after adjusting for confounders. In contrast, female participants showed a weaker positive correlation between LPA and physical function, with only the SPPB STS score, and total MoCA score showing positive associations that diminished after adjustment.

Surprisingly, PA metrics did not demonstrate significant associations with muscular strength, postural control, or global cognitive function in the total sample or among male participants. Previous studies have established a link between higher PA levels and improved physical functioning in older adults (58–61). However, SB is prevalent among those in long-term care, primarily due to frailty, comorbidities, and reduced independence (62, 63). Contrary to our hypothesis, SB only weakly correlated with SPPB STS scores and FRAIL-NH scores across the population. This unexpected finding may stem from a floor effect in SB measurement; individuals in this cohort are already highly sedentary, rendering variations in SB less impactful on physical function measures. Moreover, SB may not be as detrimental in this frail population, as their baseline physical function is already low, and further SB might not noticeably worsen their performance.

Interestingly, our results indicate that longer LPA durations are significantly associated with better physical performance, as evidenced by higher SPPB scores, faster gait speeds, and quicker STS times. This suggests that, within this frail population, LPA may be more beneficial and a better indicator of overall health than SB or MVPA. These findings are consistent with previous research showing that LPA is linked to improved health outcomes in older adults, and is more appealing and accessible for inactive, high-risk populations compared to MVPA (64, 65). Given that LPA often involves incidental activity (66), these results underscore the potential role of facility design in promoting LPA and enhancing physical function.

Consistent with earlier findings, male participants outperformed females in most physical performance tests and exhibited greater muscle strength (31, 49, 67). Although initial multiple regression analysis for the total population did not yield significant associations for some outcomes, such as the total MoCA scores, we still explored sex-specific models to uncover potential associations. Sex differences were also evident in the relationship between PA measures and physical function in this study, supporting prior research (68, 69). Stronger associations between LPA and physical function were demonstrated in male participants, while in females, LPA only weakly correlated with the SPPB STS score before adjusting for confounders. This discrepancy may be due to the generally poorer physical function observed in female participants compared to males in our study. Additionally, we noted a significant correlation between LPA and global cognitive function in females, suggesting that LPA might help mitigate the decline in cognitive function in this population. Previous evidence indicated that higher levels of PA are necessary to induce sufficient neuroplastic changes and cognitive benefits (70). Engaging in more intense or prolonged PA could enhance cerebral blood flow, neurogenesis, and synaptic plasticity, all critical for cognitive function (71). However, given the very low PA levels and severe mobility impairments observed in our participants (72), LPA appears to be the most feasible form of activity for this group.

Moreover, we did not observe significant correlations between PA metrics and muscular strength, which contrasts with findings in community-dwelling older adults from different countries (73, 74). Cultural differences, variations in PA levels, and disparities between living environments (e.g., living in long-term care versus independent living) may contribute to these inconsistencies (72). It is also essential to consider whether cross-sectional measurements of PA reflect current activity levels or provide insight into past behaviors. Cross-sectional data capture PA at a single time point, potentially failing to represent long-term PA habits or variations over time. Individuals who are currently active might have been sedentary in the past, or vice versa, and such variations can influence the observed relationships between PA and physical and cognitive function. While cross-sectional measurements provide a snapshot of current PA, they may not fully capture the cumulative effects over a lifetime. Longitudinal studies are necessary to elucidate how sustained PA influences physical and cognitive functions over time.

### 4.1 Implications of the present findings

The findings of this study underscore the importance of promoting PA to prevent declines in physical function and cognitive function among older adults living in long-term care. Our results suggest that future PA interventions should be sex-specific. For male residents, the focus could be on mobility-enhancing activities, as LPA was strongly correlated with better performance on mobility tests. Interventions could aim to increase LPA through daily walking routines, gait training, and task-based exercises. For female residents, although the correlation between LPA and physical function was weaker, the link with cognitive function was stronger. Therefore, interventions for females should emphasize cognitively stimulating activities combined with LPA, such as mind–body exercises (e.g., Tai Chi, yoga) and tasks promoting coordination and balance while engaging cognitive processes. Furthermore, longitudinal studies are needed to assess the long-term effects of PA interventions on physical and cognitive outcomes in this frail group. Understanding the role of PA in maintaining physical and cognitive function can inform tailored interventions to improve the overall well-being of long-term care residents.

### 4.2 Study strengths and limitations

The primary strength of this study is that it is the first of its nature in Hong Kong to examine objectively measured PA, physical function, and global cognitive function, as well as their associations in older adults residing in long-term care. Moreover, our study considered various potential confounders, including education level, mobility aid used, frailty status, and facility site, in addition to the common factors such as age and BMI. Despite the objective measurements and rigorous methodologies, including tri-axial accelerometers and comprehensive physical function assessments, we acknowledge the following limitations. First, the missing PA data due to non-cooperation, physical inadmissibility, and instrument malfunction resulted in a relatively small sample size, which may mask some potential relationships, and affect the generalizability of our findings. Our results may not be applicable to other settings or populations due to the unique characteristics of older adults in long-term care in Hong Kong. The cultural, social, and health-related aspects specific to this group could influence the outcomes. Additionally, we relied on care home staff to measure body height and weight considering comfortlessness of residents when removing shoes and personal items during assessment. This approach may have introduced biases. Lastly, the cross-sectional design restricts causal inferences regarding the relationships between PA and physical and cognitive function.

## 5 Conclusion

Our research addresses a gap in the literature by revealing associations between objectively measured PA and physical function and global cognitive function in older adults residing in long-term care. Our results highlight significant associations between LPA and better physical function, with sex-specific differences in these relationships. In males, LPA was strongly associated with better mobility, while in females, LPA was weakly linked to physical function but strongly correlated with global cognitive function. These findings suggest that sex-specific PA interventions are necessary to optimize the benefits of LPA in this population.

## Data Availability

The raw data supporting the conclusions of this article will be made available by the authors without undue reservation.
