# Supplementary material for: Sex differences in the associations of accelerometer-determined physical activity with physical and cognitive function in older adults living in long-term care
Source: Front Public Health. 2024 Nov 11;12:1446286. doi: 10.3389/fpubh.2024.1446286 (PMC11586375; doi:10.3389/fpubh.2024.1446286)
Supplement: Supplementary file 1 [file Data_Sheet_1.pdf]

Montreal Cognitive Assessment Hong Kong Version (HK-MoCA)
蒙特利爾認知評估香港版

姓名:
教育年數:
性別/年齡:　　日期:

視覺空間/執行功能

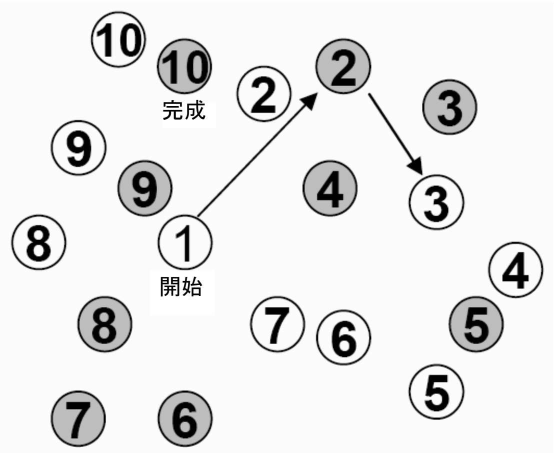

[ ]

複製圖形

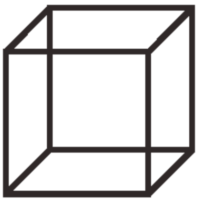

[ ]

畫時鐘 (十一點十分)
(3 分)

[ ] 輪廓　[ ] 數字　[ ] 時分針

分數 ___/5

命名

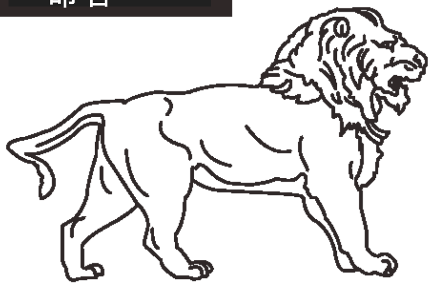

[ ]

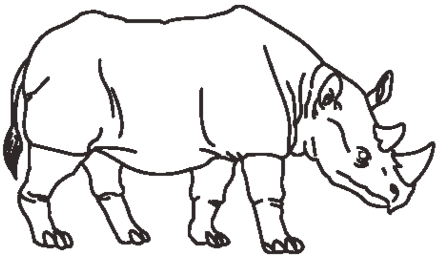

[ ]

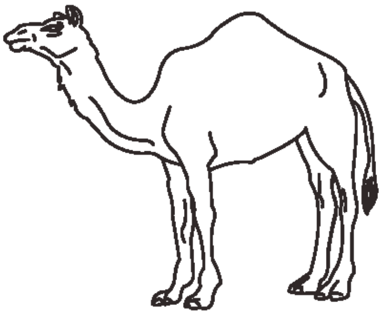

[ ]

___/3

記憶

讀出詞語再由病者重複
以上步驟做兩次
5分鐘後回憶

	面孔	絲絨	教堂	雛菊	紅色
第一次嘗試					
第二次嘗試					

不用計分

專注

讀出數字 (每秒一個)
病者須把數字向前重複 [ ] 2 1 8 5 4
病者須把數字向後重複 [ ] 7 4 2
___/2

讀出數字: 當數字 '1' 出現時病者必須用手敲打桌面
(如≥2錯誤便不給予分數)
[ ] 5 2 1 3 7 4 1 1 8 0 6 2 1 5 1 7 4 5 1 1 1 4 1 7 0 5 1 1 2
___/1

由100開始連續 7減算　[ ] 93　[ ] 86　[ ] 79　[ ] 72　[ ] 65
4 或 5 個正確減算得 **3 分**, 2 或 3 個正確得 **2 分**, 1個正確得 **1 分**, 沒有正確得 **0 分**
___/3

語言

重複: 姨丈買魚腸 [ ]
西施四十四歲 [ ]
___/2

流暢: 一分鐘內能說出的動物名稱的數目 [ ] (N ≥ 11 個名稱)
___/1

抽象

相似點: 例如: 香蕉 – 橙 =生果　[ ] 火車 – 單車　[ ] 手錶 – 間尺
___/2

延遲記憶

須回憶詞語 不可給提示	面孔 [ ]	絲絨 [ ]	教堂 [ ]	雛菊 [ ]	紅色 [ ]	分數只給予沒有提示的正確回憶
類目提示 (見下表)						
多項選擇 (見下表)						

___/5

定向

[ ] 日　[ ] 月　[ ] 年　[ ] 星期　[ ] 地點　[ ] 地區
___/6

© Nasreddine MD
Hong Kong Version updated 26 Oct 2018
Adapted by Wong A and Mok V
http://www.mocatest.org

延遲記憶備註表

	**類目提示**	**多項選擇**
面孔	身體的一部分	鼻哥、面孔、手
絲絨	紡織品的一種	牛仔布、棉花、絲絨
教堂	建築物的一種	教堂、學校、醫院
雛菊	花的一種	玫瑰、雛菊、鬱金香
紅色	一種顏色	紅色、藍色、綠色

總分 ______/30

百分位分類 (見後頁)

☐ >16th　≤☐16th　≤☐7th　≤☐2nd
